# Prognostic value of brain natriuretic peptide vs history of heart failure hospitalization in a large real‐world population

**DOI:** 10.1002/clc.23468

**Published:** 2020-09-19

**Authors:** Michael R. Zile, Akshay S. Desai, Rahul Agarwal, Rupinder Bharmi, Nirav Dalal, Philip B. Adamson, Alan S. Maisel

**Affiliations:** ^1^ Medical University of South Carolina and Ralph H. Johnson Veterans Administration Medical Center Charleston South Carolina USA; ^2^ Cardiovascular Division Brigham and Women's Hospital Boston Massachusetts USA; ^3^ Abbott Global Data Science and Analytics Sylmar California USA; ^4^ Division of Cardiovascular Medicine University of California San Diego California USA

**Keywords:** B‐type natriuretic peptide, heart failure, hospitalization, mortality

## Abstract

**Background:**

In heart failure (HF) patients, both natriuretic peptides (NP) and previous HF hospitalization (pHFH) have been used to predict prognosis.

**Hypothesis:**

In a large real‐world population, both NP levels and pHFH have independent and interdependent predictive value for clinical outcomes of HFH and all‐cause mortality.

**Methods:**

Linked electronic health records and insurance claims data from Decision Resource Group were used to identify HF patients that had a BNP or NT‐proBNP result between January 2012 and December 2016. NT‐proBNP was converted into BNP equivalents by dividing by 4. Index event was defined as most recent NP on or after 1 January 2012. Patients with incomplete records or age < 18 years were excluded. During one‐year follow up, HFH and mortality rates stratified by index BNP levels and pHFH are reported.

**Results:**

Of 64 355 patients (74 ± 12 years old, 49% female) with available values, median BNP was 259 [IQR 101‐642] pg/ml. The risk of both HFH and mortality was higher with increasing BNP levels. At each level of BNP, mortality was only slightly higher in patients with pHFH vs those without pHFH (RR 1.2 [95%CI 1.2,1.3], *P* < .001); however, at each BNP, HFH was markedly increased in patients with pHFH vs those without pHFH (RR 2.0 [95%CI 1.9,2.1], *P* < .001).

**Conclusion:**

In this large real‐world heart failure population, higher BNP levels were associated with increased risk for both HFH and mortality. At any given level of BNP, pHFH added greater prognostic value for prediction of future HFH than for mortality.

## Abstract 2

In heart failure (HF) patients, both natriuretic peptides (NP) and previous HF hospitalization (pHFH) have been used to predict prognosis. However, this association has not been reported over a wide range of NP levels with and without pHFH for clinical outcomes of HFH and all‐cause mortality in a large real‐world population. The current study showed that in a large real‐world heart failure population, higher BNP levels were associated with increased risk for both HFH and mortality. At any given level of BNP, pHFH added greater prognostic value for prediction of future HFH than for mortality.

## 1 INTRODUCTION

Clinical and laboratory metrics capable of accurately predicting mortal (all‐cause mortality) and morbid (heart failure hospitalization) event rates serve several critical roles. For example, they can be used to facilitate assessment and management of health care resources for defined populations, they can play a pivotal role in planning sample size calculations for randomized clinical trials, they can enhance our ability to define disease phenotype, and define populations that are responsive to existing and novel management strategies.

Among the clinical and laboratory metrics that have been shown to predict morbidity and mortality in chronic heart failure patients (HF), two have demonstrated the most promise: previous HF hospitalizations (HFH) and natriuretic peptides (NP), particularly B‐type natriuretic peptide (BNP).^1^, ^2^, ^3^, ^4^, ^5^, ^6^, ^7^ To date, the experimental designs used to examine the utility of these metrics have had several limitations. For example, previous studies have had limited sample sizes, been constrained by exclusion of co‐morbidities, had short follow‐up periods, and not reflected a non‐selected “real world” population. Furthermore, previous studies have examined binary cut‐off values of BNP, above vs below median values, data division in tertiles or quartiles. They have not examined and compared HF with a reduced ejection fraction (HFrEF) vs HF with a preserved EF (HFpEF). While both HFH and NP levels are consistently associated with greater risk for morbidity and mortality in patients with HF, the extent to which these two parameters in combination help to stratify risk compared with either parameter alone remains unclear.

Accordingly, the purpose of this study was to define the rate of HFH and all‐cause mortality that occurs over a wide range of BNP in an unselected real world population of patients with HF and determine whether the addition of a previous HFH independently altered the morbidity and mortality rates predicted by BNP in this population.

## 2 METHODS

### 2.1 Data source

Data were extracted from a US wide, real world, data repository from the Decision Resources Group. This data links medical encounter claims, prescription claims, and electronic medical records (EMR) to provide longitudinal patient‐level data covering the majority of the US healthcare system. The data were collected from the four largest clearing houses in US and covers over 1.8 million health care providers and 300 million patients. These data consist of records for tests ordered, test results, diagnoses, comorbidities, medications, therapies, patient demographics, healthcare encounters, and death. Encounters recorded in the insurance claims included in‐patient/out‐patient/emergency/urgent‐care healthcare provider visits, hospital admissions, and nursing home, rehabilitation facility, or hospice encounters.

### 2.2 Cohort design and eligibility

HF patients were identified from the data repository that had at least one measurement of BNP or NT‐proBNP test result available between 1 January 2012 and 31 December 2016. In these patients, the first BNP or NT‐proBNP result on or after 1 January 2012 was used as index event. An individual was identified as being a HF patient based on their ICD‐9‐CM or ICD‐10‐CM diagnoses for HF (Table S5) in any field in the insurance claims or EMR data on or before index event. Those with age <18 years, discontinuous insurance enrollment or missing encounter types were excluded. The status of insurance enrollment was determined using encounters with the healthcare system that resulted in an insurance claim, which included medication refill, healthcare provider visits admissions, nursing home, rehabilitation, or hospice.

### 2.3 Demographics, comorbidities, and follow‐up

Age at the index event and gender were retrieved from the EMR and healthcare encounter claims. Additionally, elixhauser comorbidities at index event were computed [Quan 2005, Moore 2017] using presence of corresponding ICD‐9‐CM or ICD‐10‐CM codes anytime on or before index event. For details on the coding and comorbidity derivations Table S6. After the index event, patient data were collected for 365 days, or until date of death, or 4 months after the last date in prescriptions/lab tests/health‐care encounters, whichever length of follow up was shorter.

The data in our analysis consisted of both electronic medical records (EMR) and insurance claims; hence, we believe that healthcare utilization data are well represented in this manuscript. We limited the history to 1 year because of the following reason. The prognostic value of a previous HFH changes over time after hospital discharge. The chances of a repeat HFH occurring after each HFH decreases the farther away you get from the discharge date. Multiple studies have demonstrated that the highest chance of a repeat HFH occurs within 1 year after discharge. It is for this reason that we limited the assessment of previous year HFH to 1 year. We only included a HFH event that occurred in the 365 days prior to the NP testing to classify a patient as having previous‐HFH or not. Making this choice allowed the HFH to have the highest possible effect on outcomes.

### 2.4 Retrieval of lab values (NT‐proBNP or BNP)

BNP and NT‐proBNP values were retrieved from EMR records. Lab values were identified as being NP related by looking for LOINC code (Logical Observation Identifiers Names and Codes), test name and/or panel name representing NP. A total of 1049 unique combinations of test name and panel names were used to identify BNP or NT‐proBNP test. Table S8 lists top 30 combination of test and panel names along with frequency of their occurrence. All tests that were ordered but not performed were removed from the analysis. NT‐proBNP readings were converted into BNP equivalents by dividing by 4.

### 2.5 Outcomes

All‐cause mortality and HFH rate were examined across seven different baseline BNP level (0‐249, 250‐499, 500‐749, 750‐999, 1000‐1249, 1250‐1499, and ≥ 1500 pg/ml at index event) over the follow‐up period. The status and date of death were retrieved from the records in both EMR and medical claims data. First, the medical claims were searched for the discharge dates with discharge status code: 20 (expired), 40 (expired at home), 41 (expired in medical facility), 42 (expired‐place unknown) [Ref: https://www.resdac.org/cms-data/variables/patient-discharge-status-code]. The status of death was determined using records from both EMR (death status flag) and medical claims data, and no approximations were made on the status of death. The discharge dates with ICD‐9‐CM and ICD‐10‐CM codes for diagnosis of death (Table S7 for the list of codes used). The date of death was determined using last‐updated‐field in the EMR and from discharge date and status from healthcare encounters. Only when these two dates were not available, we needed to approximate using the last date in prescriptions, lab tests, or encounter data.

Hospitalizations of all types were categorized as being heart failure related (HFH) or not based on presence of comprehensive list of diagnosis codes reported in Table S5. The HFHs were determined using presence of primary or admission diagnosis of HF from inpatient hospital claims, or an MS‐DRG of 291, 292, or 293. In instances wherein multiple claims were generated from a single in‐patient hospitalization event, the claim from and claim through dates were used for consolidation of a hospitalization event. Any hospitalization spanning the index event was deemed to be a pre‐index event and not counted toward the post‐index follow‐up period.

### 2.6 Sub‐group analysis

The impact of BNP on clinical outcomes was examined in patients with HFH in previous year vs those without and patients with heart failure and a reduced ejection fraction (HFrEF) vs heart failure and a preserved ejection fraction (HFpEF). In a cohort in which BMI and HF type were known, we adjusted the BNP and examined the outcomes of HFH rate and mortality rate. This adjustment was based on the Guide‐HF clinical trial, design, and rationale. BNP was adjusted by increasing it by 75 pg/ml for a HFpEF patient, and by 4.1% for every unit increase in BMI above 25 units.^8^

#### 2.6.1 Patients with HFH in previous year vs those without

Hospitalizations of all types were adjudicated as being heart failure related or not. A count of the HFH in the 365 days preceding and including the index date was used to classify patients into two groups, “no previous year HFH” and “previous year HFH”.

#### 2.6.2 HFrEF vs HFpEF

HFrEF was identified by the presence of ICD‐9‐CM code of 428.2 (systolic heart failure) or ICD‐10‐CM code I50.2 (systolic [congestive] heart failure) available in the healthcare encounters. HFpEF was identified by presence of ICD‐9‐CM code of 428.3 (diastolic heart failure) or ICD‐10‐CM code I50.3 (diastolic [congestive] heart failure). In cases where an echocardiographic test result was known and the ejection fraction was less than 40, the patient was characterized as HFrEF patient regardless of the diagnosis code.

### 2.7 Statistics

Continuous variables are presented as mean ± sd and were compared using a Student *t* test unless otherwise stated. Categorical variables are presented as n (%) and were compared using chi‐squared test. HFH are presented as 1‐year Nelson‐Aalen estimates (events/patient); which are non‐parametric estimator of the cumulative hazard function and were compared using the Anderson‐Gill modification of the Cox‐proportional hazard model for recurrent events with adjustment for age, gender, comorbidities, and previous year HFH. Mortality is presented as 1‐year Kaplan‐Meier survival estimates and were compared using Cox‐proportional hazard models with adjustment for age, gender, comorbidities and previous year HFH. In addition, HFH or mortality rates are presented as events per patient‐year, and are compared using nonparametric bootstrap methodology with replacement with 1000 iterations. The specific demographics and comorbidities used in the model are presented in Supplemental Table S1. Statistical analyses were performed in Jupyter Notebook version 4.2.1 running on Python version 3.5.3, and Anaconda version 4.1.1 (64‐bit) (https://jupyter.org/) and R Studio Version 0.99.486 running on R version 3.1.3 (https://www.rstudio.com/).

## 3 RESULTS

### 3.1 Sample size and demographics

In the Decision Resource Group real‐world data repository, 81 610 HF patients were identified that had a BNP or NTpro‐BNP test performed between January 2012 and December 2016. Only 4.8% of the NP assays were performed during hospital admission (the time duration between a hospital‐admission and discharge). The remaining 95.2% of the NP assay results were obtained outside of a hospital setting during periods of clinical stability. Of the 81 610 HF patients, 670 (<1%) patients were less than 18 years old, 15 949 (19.5%) did not have any follow up medical encounter either in the year before or after BNP test was done, and 636 (<1%) had missing information on medical encounter type and hence were excluded from the analysis (Figure S1). The final study cohort comprised of 64 355 patients.

Demographic data for the study cohort are presented in Table S1. Mean age was 74.3 ± 11.7 years, 49.3% were female, 47.6% had diabetes, and 88.1% had a history of hypertension. The median BNP (or equivalent) level was 259 [IQR 101‐642] pg/ml. Compared with patients in the lowest BNP group (0‐249 pg/ml), all higher BNP groups were older and had higher prevalence of comorbidities, such as, renal failure, cardiac arrhythmias, coronary artery disease, pulmonary circulation disorders, deficiency anemia, fluid and electrolyte disorder, valvular disease and weight loss, whereas obesity, drug, and alcohol abuse were less prevalent. Diseases, such as, hypertension, COPD, AIDS, and cancer were similarly prevalent across all BNP groups.

### 3.2 Correlations between BNP and heart failure hospitalization rate

**FIGURE 1:**
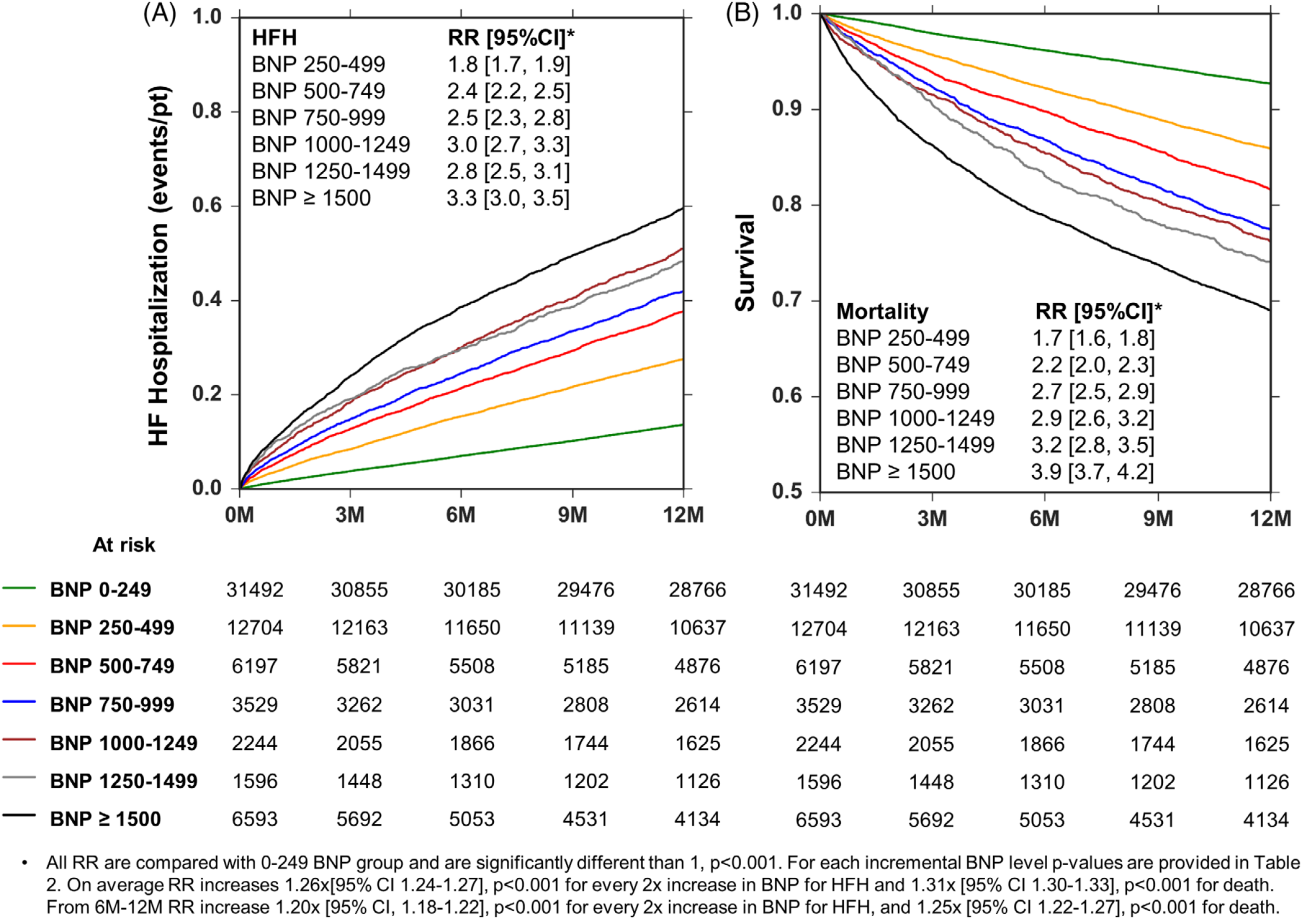
Heart failure hospitalizations (HFH) and survival stratified by B‐type natriuretic peptide (BNP) levels. Panel A, nelson aalen estimate of HFHs stratified by BNP levels. Panel B, Kaplan‐Meier estimate of survival stratified by BNP levels

The risk of HFH increased as the BNP level rose (Figure 1A. Patients with BNP ≥1500 pg/ml had highest risk of HFH (RR: 3.3; 95% CI 3.0‐3.5) followed by BNP 1000 to 1249, 1250 to 1499, 750 to 999, 500 to 749, 250 to 499 and 0 to 249 groups. Compared with BNP 0 to 249 pg/ml, the risk ratios incrementally increased with each subsequent BNP level, except group 1250 to 1499, which has non‐significant difference with group 1000 to 1249. For example, as shown in Figure 1A, the RR for HFH is 1.8 for BNP 250‐499 vs 2.4 for BNP 500‐749. The *P*‐value corresponding to this comparison is <.001. The RR for HFH is 3.0 for BNP 1000‐1249 vs 2.8 for BNP 1250‐1499. The *P*‐value corresponding to this comparison is .323. The statistical comparison of the risk of HFH between each incremental BNP group is listed in Table S2. The overall RR for HFH was 1.26 for every 2 times increase in BNP.

### 3.3 Correlations between BNP and all‐cause mortality

The risk of all‐cause death increased as the BNP level rose as shown in Figure 1B. Patients with BNP ≥1500 pg/ml had highest risk of death (RR: 3.9; 95%CI 3.7‐4.2) followed by BNP 1250 to 1499, 1000 to 1249, 750 to 999, 500 to 749, 250 to 499 and 0 to 249 pg/ml groups. Compared with BNP 0 to 249 pg/ml, the hazard ratios incrementally increased with each subsequent BNP level. For example, as shown in Figure 1B, the RR for mortality was 1.7 for BNP 250‐499 vs 2.2 for BNP 500‐749 pg/ml. The *P*‐value corresponding to this comparison was <.001. The RR for mortality was 2.9 for BNP 1000‐1249 vs 3.2 for BNP 1250‐1499 pg/ml. The *P*‐value corresponding to this comparison was 0.217. The statistical comparison of the risk of mortality between each incremental BNP group is listed in Table S2. The overall RR for mortality was 1.31 for every 2 times increase in BNP (This overall RR was derived using multivariate analysis using BNP as a continuous variable).

### 3.4 Effect of previous year HFH on correlations between BNP and subsequent HFH

**FIGURE 2:**
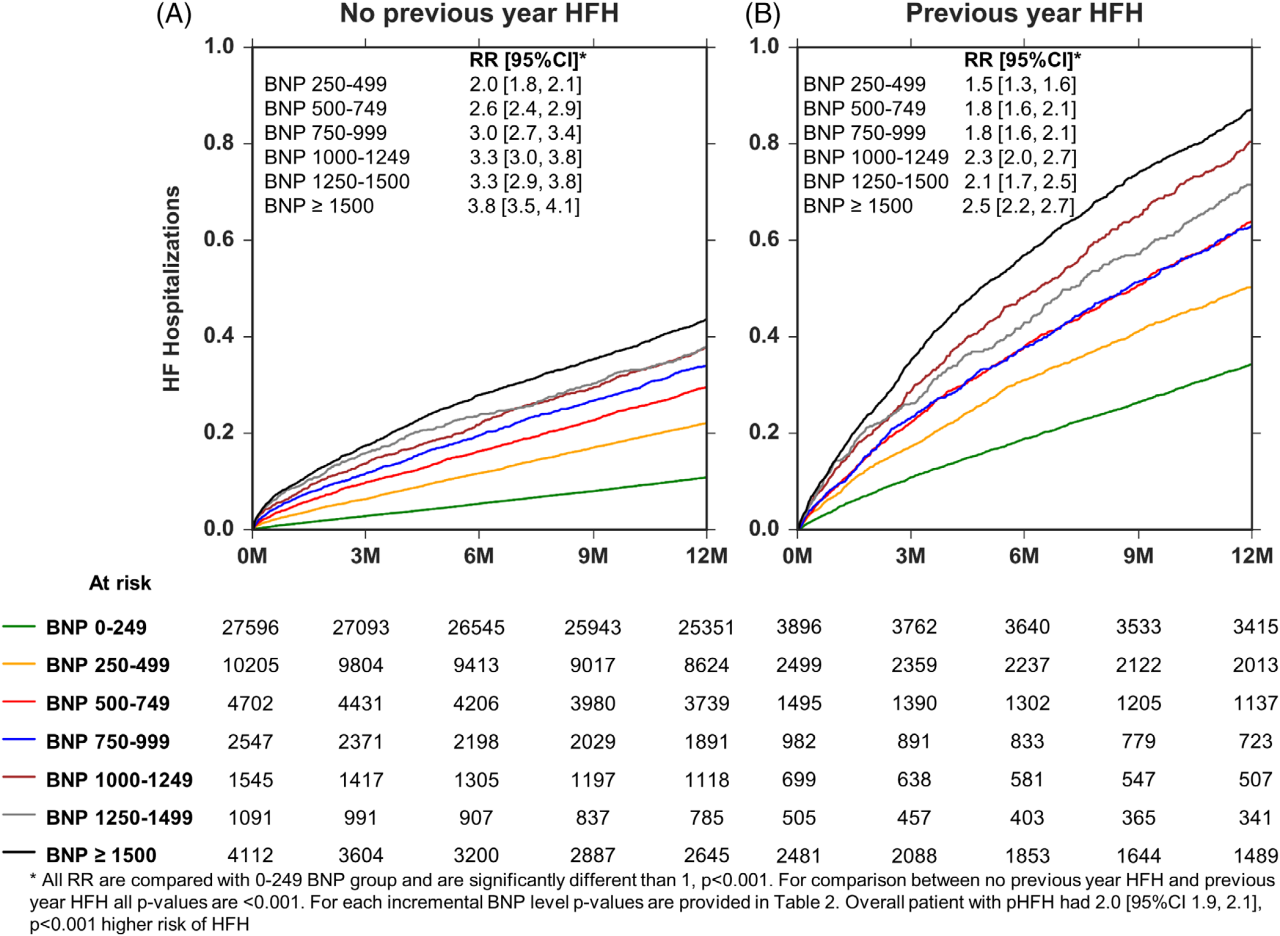
Affect of previous heart failure hospitalization (pHFH) in previous year on future HFH stratified by B‐type natriuretic peptide (BNP) levels using Nelson‐Aalen analysis. Panel A, HFH at each BNP range in patients without a pHFH. Panel B, HFH at each BNP range in patients with a pHFH

Patients with a HFH in previous year had 2.0 [95%CI‐ 1.9‐2.1]; *P* < .001 times higher risk of HFH in the subsequent year compared with those who did not have a HFH in the previous year after adjusting for age, gender, co‐morbidities and BNP‐levels. At every bracketed range of BNP, patients with a HFH in previous year had a statistically significant higher risk of HFH in the subsequent year (Figures 2 and Figure S2). For example, at a range of BNP between 0 and 249 pg/ml, the risk of HFH increased from 0.11 events/patient year in patients that had not had a previous HFH in the previous year to 0.35 events/ patient year (Figure S2) in patients that had a previous HFH in the previous year (RR = 2.7 [95%CI 2.5, 3.0], *P* < .001). Findings were similar at each bracketed range of BNP.

### 3.5 Effect of previous year HFH on correlations between BNP and subsequent all‐cause mortality

**FIGURE 3:**
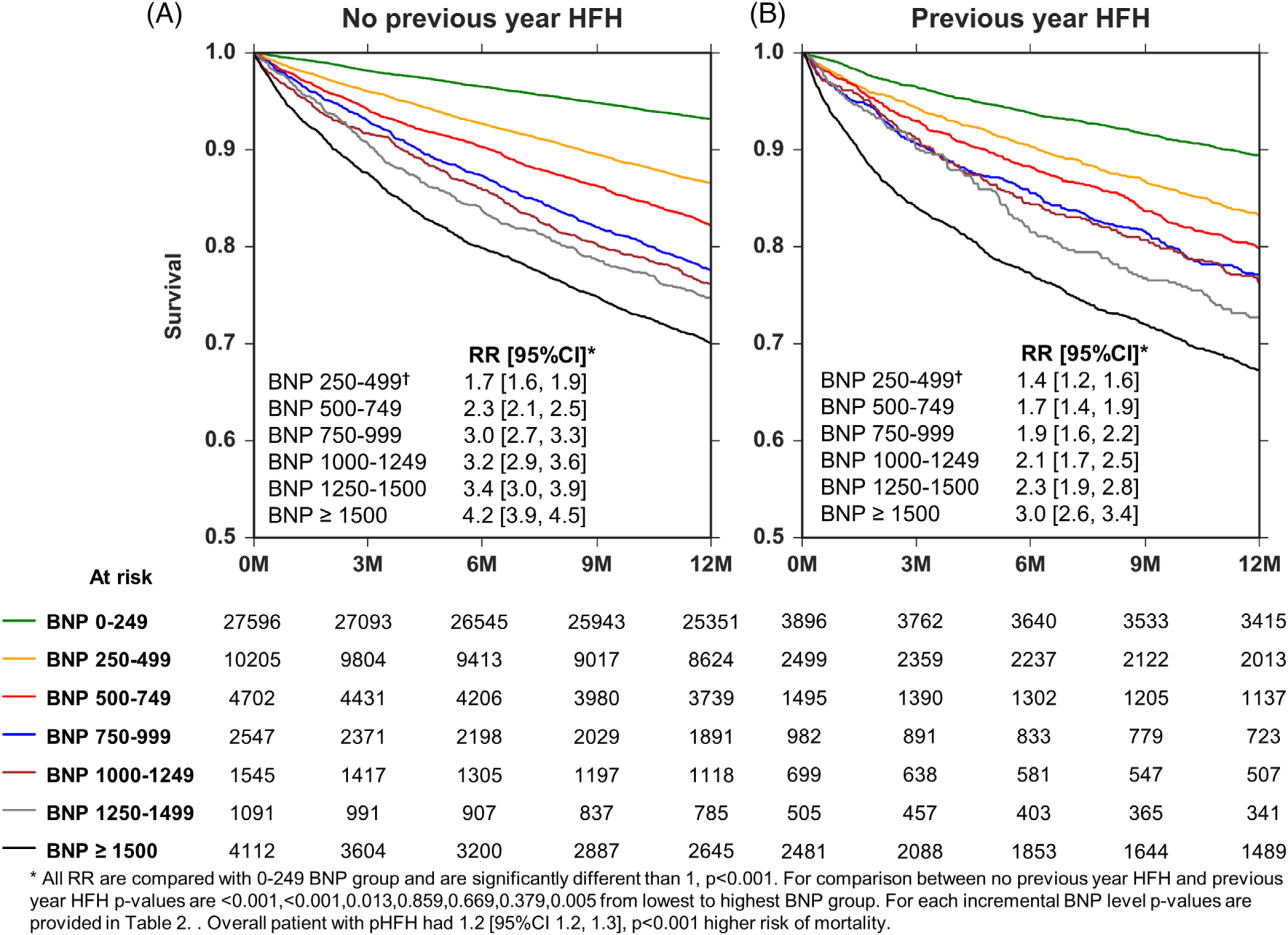
Kaplan‐Meier survival curves stratified by history of HFH in previous year and BNP levels Panel A, survival at each B‐type natriuretic peptide (BNP) range in patients without a pHFH. Panel B, survival at each BNP range in patients with a pHFH

Patients with a HFH in previous year had 1.2 [95%CI 1.2‐1.3], *P* < .001 times higher risk of all‐cause mortality in the subsequent year compared with those who did not have a HFH in the previous year after adjusting for age, gender, co‐morbidities, and BNP‐levels. In general, at every bracketed range of BNP, patients with a HFH in previous year tended to have a higher risk of all‐cause mortality in the subsequent year; however, this difference did not reach statistical difference in each case (Figure S2 and Figure 3). The differences between the effects of a previous HFH added to BNP did not have as extensive an impact on all‐cause mortality as it did on subsequent HFH. For example, at a range of BNP between 0 and 249 pg/ml, the risk of all‐cause mortality increased from 0.07 events/patient year in patients that had not had a previous HFH in the previous year to 0.11 events/patient year in patients that had a previous HFH in the previous year (RR = 1.6 [95%CI 1.5‐1.8], *P* < .001), corrected for age, gender, and comorbidities. In contrast, at a range of BNP between 650 and 999 pg/ml, the risk of all‐cause mortality increased from 0.26 events/patient year in patients that had not had a previous HFH in the previous year to 0.27 events/patient year in patients that had a previous HFH in the previous year (RR = 1.0 [95% CI 0.9‐1.2], *P* = .859), again corrected for age, gender, and comorbidities.

### 3.6 Effect of HF phenotype on correlations between BNP, HFH and all‐cause mortality

**FIGURE 4:**
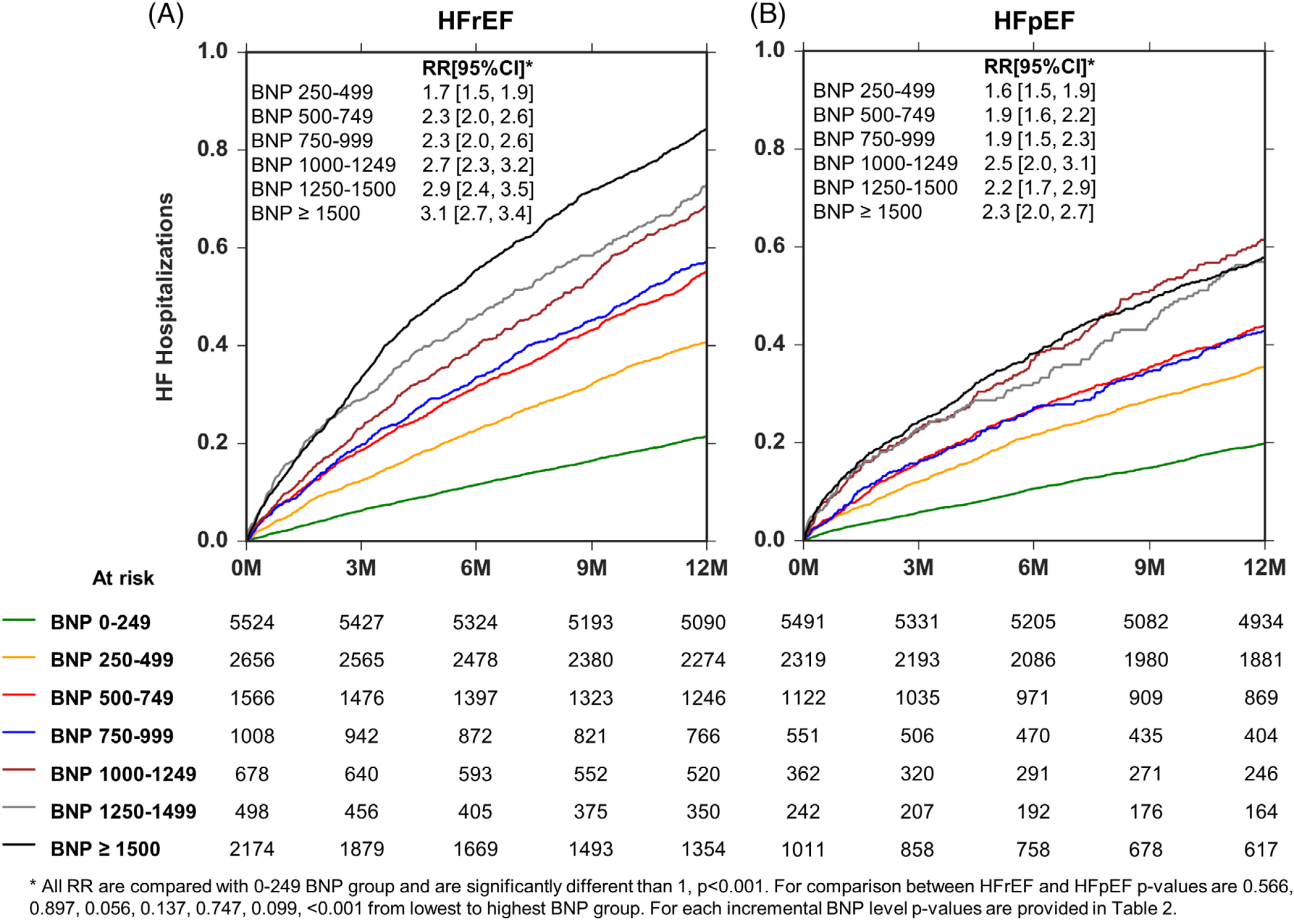
Heart failure hospitalizations (HFH) stratified by heart failure phenotype and B‐type natriuretic peptide (BNP) levels using a Nelson‐Aalen analysis. Panel A, HFH at each BNP range in patients with heart failure and a reduced ejection fraction (HFrEF). Panel B, HFH at each BNP range in patients with heart failure and a preserved ejection fraction (HFpEF)

**FIGURE 5:**
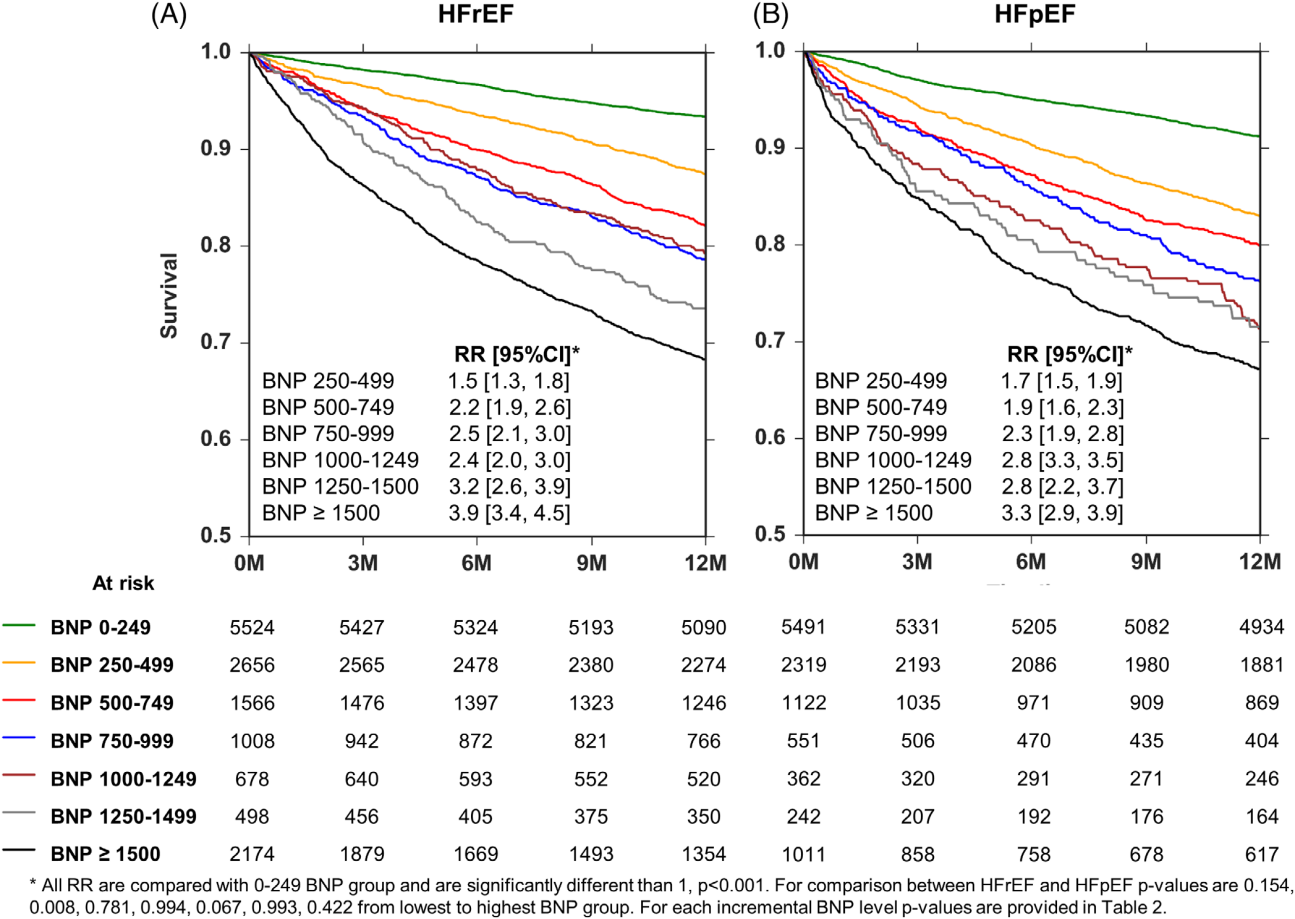
Kaplan‐Meier survival curves stratified by heart failure phenotype and B‐type natriuretic peptide (BNP) levels. Panel A, Survival at each BNP range in patients with heart failure and a reduced ejection fraction (HFrEF). Panel B, Survival at each BNP range in patients with heart failure and a preserved ejection fraction (HFpEF)

Of the 64 355 patients examined, 39 153 could not be HF determined, 14 104 were classified as HFrEF and 11 098 as HFpEF (Figure S3). The risk of HFH and the risk of all‐cause mortality increased in both HFrEF and HFpEF patients as a function of increasing BNP (Figures 4 and 5). For any given range of BNP, the risk ratio of HFH was generally lower in the HFpEF patients compared with the HFrEF patients. For example, at a range of >1500 pg/ml, risk ratio for HFH was 3.1 (0.89 events per year) in HFrEF vs 2.3 (0.62 events per year) in HFpEF, *P* < .001 as compared with 0‐249 pg/ml BNP range. However, these differences did not reach statistical significance in all of the BNP brackets (Table S2). By contrast, for any given range of BNP, the rate of all‐cause mortality was similar in the HFpEF patients compared with the HFrEF patients (Figure 5).

### 3.7 Effect of age on BNP and correlations between BNP and HFH and all‐cause mortality

Overall BNP levels increased with the increasing age brackets examined (Figure S1). Additionally, at every age, the risk of HFH and all‐cause mortality increased as a function of increasing BNP brackets (Figure S5 and S6).

### 3.8 Outcomes for BNP adjusted for LVEF, BMI, and renal function

For any given level of heart failure severity, BNP was altered in the presence of Afib, LVEF and other factors. Risk ratios for HFH and mortality were assessed using BNP levels that were adjusted for BMI and LVEF in a subgroup of 3590 patients in whom both were known. BNP was increased by 75 pg/ml for a HFpEF patient, and increased by 4.1% for every unit increase in BMI above 25 units. The outcomes indicate the same trend across the adjusted BNP strata (Table S3) when compared to unadjusted BNP analyses.

In the current study, outcomes were adjusted for age, gender, comorbidities, and previous year HFH. Among the comorbidities examined was renal failure status. Relative risk for death was 1.17 [1.11‐1.22], *P* < .05 in patients with history of renal disease, and was 1.20 [1.15‐1.25], *P* < .05 for HFH. These data are similar to previous publications.^9^

### 3.9 Outcomes according to BNP vs NT‐proBNP

The index NP tests were a combination of BNP and NT‐proBNP, such that 64.4% of index test results were from BNP, and the remaining 35.6% were from NT‐proBNP. The relationship of NP levels and outcomes remained similar irrespective of the test type (BNP vs NT‐proBNP) used in the analysis.

## 4 DISCUSSION

In heart failure patients, both brain natriuretic peptide and previous HF hospitalization predict prognosis.^1^, ^2^, ^3^, ^4^, ^5^, ^6^, ^7^ However, this association has not been reported over a wide range of BNP levels and analyzed in patients with and without prior HFH for both HFH and all‐cause mortality in a large, real‐world population. Data from the current study support several clinically relevant findings. Over a very wide range of BNP, subdivided into seven ranges, the risk of HFH increased nearly 26% for every doubling of the BNP value. These differences persisted over time, with risk in 6 to 12 months 20% higher. The same findings were true for all‐cause mortality. The risk of all‐cause mortality increased nearly 31% for every doubling of the BNP value. When a history of a previous HF hospitalization was added to the risk model, there was an important added value in predicting future HFH, but less added value in predicting all‐cause mortality. These finding were consistent across HF phenotypes defined by EF and age. The risk of HFH and the risk of all‐cause mortality increased in both HFrEF and HFpEF patients and at every age bracket as a function of increasing BNP. It is important to note that, these findings were obtained in a large, completely “unselected” (real‐world) population with no exclusions for co‐morbidities or any other factors that would routinely be done in RCTs.

This study represents the largest cohort report showing the association of natriuretic peptides (NP) and heart failure hospitalizations (HFH) as predictors of outcomes in HF patients. Very specifically, this study showed that higher BNP levels were associated with increased risk for both HFH and mortality. The unique observation from this study is at any given level of BNP, previous HF hospitalization added greater prognostic value for prediction of future HFH than for mortality. Similarly, regardless of whether the patient had HFH in previous year, the knowledge of BNP added greater prognostic value about future HF hospitalization and, most importantly, about mortality.

### 4.1 Practical applications

The data and analysis provided by this study should help to facilitate assessment and management of health care resources for defined populations, play a pivotal role in planning sample size calculations for randomized clinical trials, enhance our ability to define disease phenotype, and define populations that are responsive to existing and novel management strategies.

For example, one of the most difficult decisions made in the design of clinical trials relates to the oppositional effects of using BNP to enhance specificity and increase event rates vs resultant effects on rates of recruitment. Within eligibility criteria, the higher the required BNP and the additional requirement of prior HFH usually results in a significant increase in the number of patients that will be “screened out” as a potential participant and will result in a slower recruitment rate for a study. A proper balance between estimated event rates and recruitment rate may be achieved by using the data in the current study. If the primary endpoint is all‐cause mortality, the eligibility criteria may focus more heavily on BNP values because the requirement of prior HFH does not appear to predict mortality to as significant a degree. However, if HFH rate is the primary endpoint, adding the requirement of prior HFH will lower the needed BNP level for eligibility. In either circumstance, precise numbers may be useful from the data provided in this study.

In addition, the current data suggest that BNP and prior HFH data are practical predictors of events in both HFrEF and HFpEF and at all age groups. These data also point to the facts that the rate of HFH is lower at any given BNP in patients with HFpEF and with increasing age. Uniquely, the rates of all‐cause mortality at any given BNP are comparable for HFpEF vs HFrEF.

### 4.2 Comparisons with RCT or epidemiology based data

The rates of HFH and all‐cause mortality obtained in the current study are comparable to both the data bases published in RCTs and epidemiology studies. Examples of data from several recent clinical trials that have incorporated BNP and NT‐proBNP in the inclusion criteria, are shown in Table S4.^10^, ^11^, ^12^, ^13^, ^14^, ^15^, ^16^, ^17^

### 4.3 Limitations

Several limitations to the current data and analysis are noteworthy. The data from this study cannot be used to predict response to therapies, particularly novel therapies. There is insufficient data to compare the relative value of BNP vs NT‐proBNP. For any given level of heart failure severity, BNP is altered by the presence of Afib, BMI, and other factors. In a limited dataset, we have addressed the impact of LVEF and BMI via a BMI adjusted BNP analysis (Table S3), but are unable to assess the impact of atrial fibrillation. These co‐morbid factors were not examined within the context of this study or placed into a multifactorial analysis to adjust or normalize for the HFH or mortality rates. These analyses are beyond the scope of this study.

The influence of the number of prior HF hospitalizations on subsequent cardiovascular events in patients with reduced or preserved EF is important and has been studied in a number of clinical trials including CHARM, I‐Preserve and others^18^ Belle et al. demonstrated that history of an acute heart failure event is a powerful predictor of adverse cardiovascular outcomes, and the time between the last HFH and enrollment was a powerful predictor of subsequent event rates. However, given the focus and methodological limitations of the current analysis, we are not able to perform this analysis. In the current study design, we were able to adjust for age, gender, co‐morbidities, and previous year HFH as discrete variables and did not account for the number of events and time between the last HF hospitalization and the NP value. We acknowledge that this is a worthwhile analysis that will await further study.

While both BNP and NT‐proBNP are widely used to aid diagnosis, assess the effect of therapy, and predict outcomes in heart failure, there is no clear consensus to guide the conversion between them.^19^ However, there are examples of conversion factors chosen for recent randomized clinical trials that were used to choose and justify a conversion factor of 4 times BNP, NT‐proBNP as used in the current analysis. For example, the eligibility criteria in PARADIGM‐HF included elevated natriuretic peptides: BNP ≥150 pg/ml or NT‐proBNP ≥600 pg/ml (for patients with HF hospitalization within 12 months, BNP ≥100 pg/ml or NT‐proBNP ≥400 pg/ml), that is, a factor of 4. A similar conversion factor was chosen for the ongoing trial Guide‐HF (Lindenfeld et al.^22^). Because the conversion factor likely varies with disease states, with and without atrial fibrillation, with increasing age and decreasing renal function Rorth et al.^19^ concluded that “there is no single, simple, conversion ratio of NTproBNP to BNP and factors, such as, atrial fibrillation, age, and renal function need to be taken into account.” There are alternative conversion formulas proposed by Kasahara et al.^20^ and Yeo et al.^21^ that incorporate age, body mass index, hemoglobin, renal function, sex, and atrial fibrillation; however, these variables were not available in the context of the current study. Whether alternative conversions factors with different numerical values or with incorporation of additional variables would change the conclusions of this analysis must await further study.

The use of ICD codes to designate patients as having HFrEF vs HFpEF has significant limitations because it is not obligatorily linked to an objective measure of ejection fraction. It does; however, take advantage of the fact that the clinical provider who knows the patient the best is the one that can impact the choice the ICD code. For these reasons, our analysis reported in the manuscript used a conservative approached wherein we classified a patient as HFrEF, if either they had a diagnosis of systolic HF (ICD code) OR their EF was <40. Where, a patient was classified as HFpEF when their claims carry a diagnosis of diastolic HF and their EF was not <40 if it was known.

## 5 CONCLUSION

In a large real‐world heart failure population, higher BNP levels were prognostic of both future HFH and mortality whereas at any given level of BNP, previous HFH added prognostic value to a greater degree for prediction of future HFH than for mortality.

## CONFLICT OF INTEREST

Dr Zile serves as a consultant to Abbott for Device Development and is a member of the executive steering committee for the Abbott funded clinical trial Guide‐HF. Dr Desai has received research grant support from Novartis, AstraZeneca, and Alnylam as well as honoraria for consulting from Abbott, AstraZeneca, Alnylam, Boston Scientific, Boehringer Ingelheim, Biofourmis, Corvidia, DalCor Pharma, Merck, Novartis, Relypsa, and Regeneron. Rahul Agarwal, Rupinder Bharmi, Nirav Dalal and Philip B. Adamson are salaried employees of Abbott. Dr Maisel has received honoraria for consulting from Abbott, Quidel, and is co‐founder of Brainstorm Medical.

DATA SOURCE

Note: Claims = Administrative claims data. For each healthcare encounter, the billing data are submitted as a claim, and consists of the admission date, discharge date, type of encounter (in‐patient, outpatient), ICD‐9‐CM/ICD‐10‐CM Diagnoses codes and Procedure codes.

## Supporting information


**Figure S1**: Consort Diagram.
**Figure S2**: 1 year mortality rates and heart failure hospitalization rates (HFH) rates across BNP levels with and without previous HFH (pHFH). This analysis examines the statistical difference at each BNP range between mortality with vs without pHFH (black bars) and between HFH with and without pHFH (red bars). For comparison of mortality between with‐ and without‐ pHFH, *P*‐values are <.001, unless otherwise mentioned and are color coded (black type). For comparison of HFH between with‐ and without‐ pHFH, all *P*‐values are <.001. For comparisons between each incremental BNP level, *P*‐values <.001, unless otherwise mentioned and are color coded (black type for mortality, red type for HFH.
**Figure S3**: Consort Diagram for HFpEF vs HFrEF Cohort sub‐analysis
**Figure S4**: Correlations between age and BNP levels
**Figure S5**: Heart failure hospitalizations (HFH) rates stratified by age and BNP levels
**Figure S6**: Mortality rates stratified by age and BNP levels

**Table S1**: Patient characteristics
**Table S2**: *P*‐values for each incremental BNP group comparison.
**Table S3**: Risk ratios for adjusted BNP across different groups.
**Table S4**: Estimated HF hospitalization rates and mortality rates from HF studies
**Table S5**: Billing codes used to identify diagnosis for heart failure
**Table S6**: Diagnoses Codes Used to Identify Comorbidities
**Table S7**: Diagnoses Codes Used to Identify Death
**Table S8**: Illustration of panel names and test names associated with BNP, NT‐proBNP for top 30 occurrences

## Data Availability

Research data are not shared.
