# Supplementary material for: Prognostic value of brain natriuretic peptide vs history of heart failure hospitalization in a large real‐world population
Source: Clin Cardiol. 2020 Sep 19;43(12):1501–10. doi: 10.1002/clc.23468 (PMC7724209; doi:10.1002/clc.23468)

Supplemental Figure 1

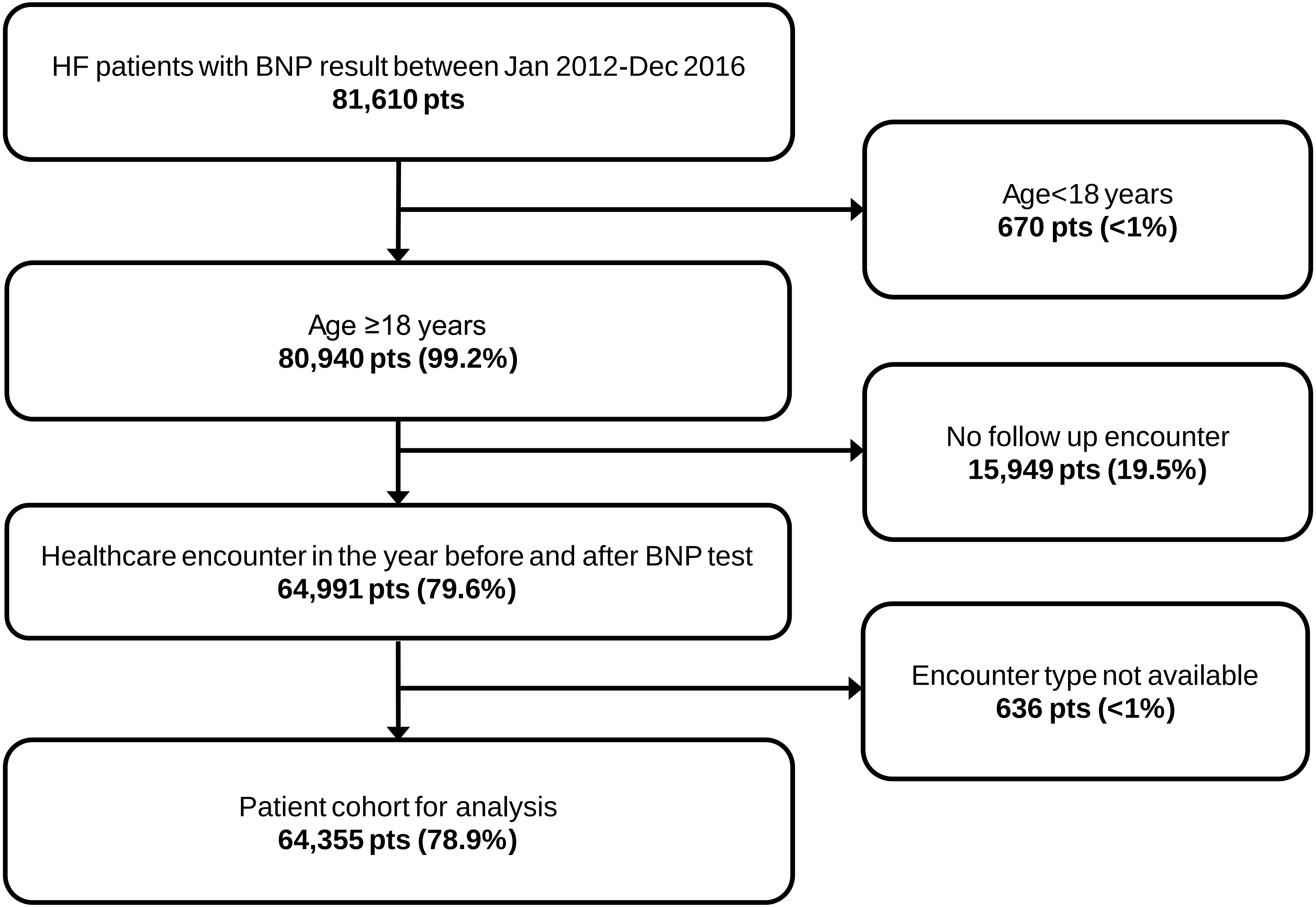

Supplemental Figure 2

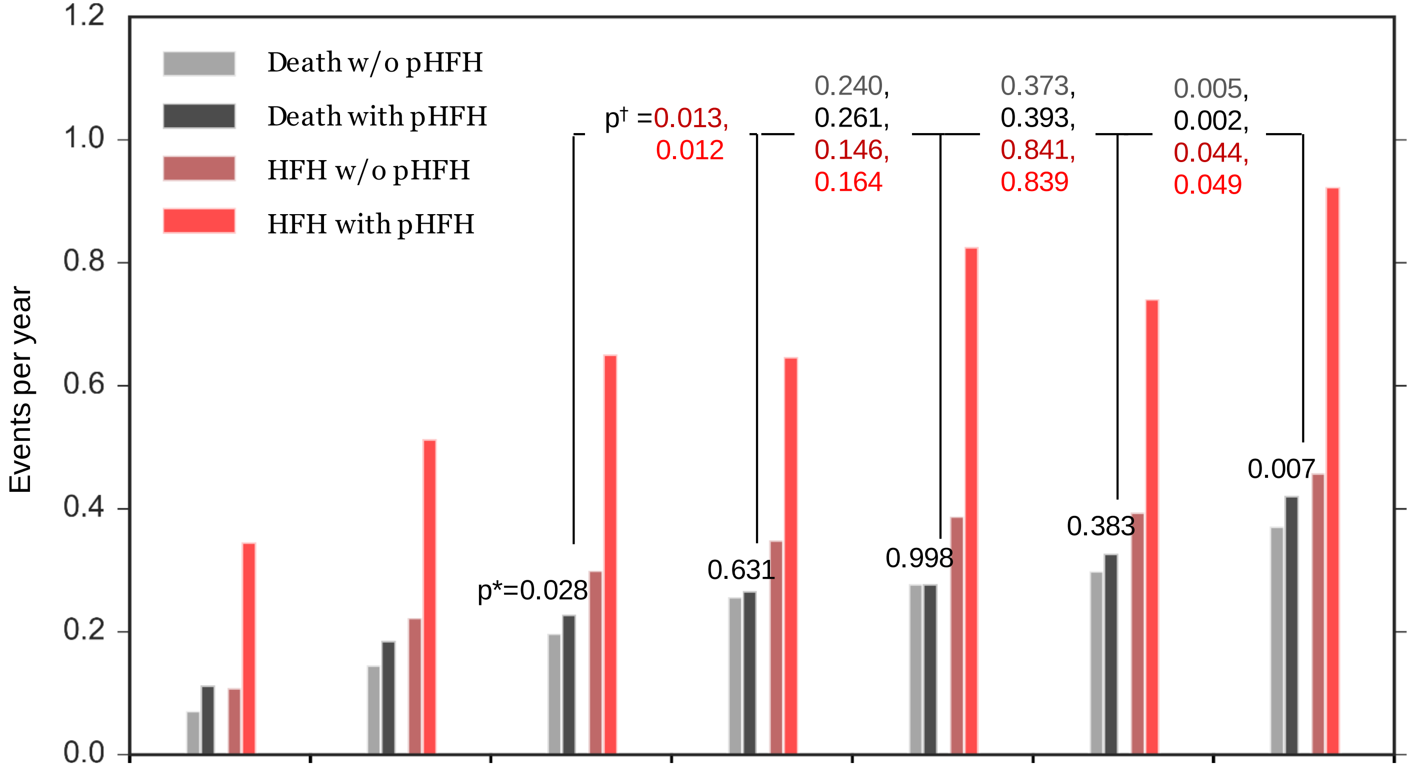

BNP (pg/ml)	0-249	250-499	500-749	750-999	1000-1249	1250-1499	≥ 1500
Pts w/o pHFH	27596	10205	4702	2547	1545	1091	4112
Pts with pHFH	3896	2499	1495	982	699	505	2481
Death w/o pHFH	0.07	0.15	0.20	0.26	0.28	0.30	0.37
Death with pHFH	0.11	0.18	0.23	0.27	0.28	0.33	0.42
HFH w/o pHFH	0.11	0.22	0.30	0.35	0.39	0.39	0.46
HFH with pHFH	0.35	0.51	0.65	0.65	0.83	0.74	0.92

* For comparison between with- and w/o- pHFH. P-values <0.001, unless otherwise mentioned and are color coded.
† For comparison between each incremental BNP level. P-values <0.001, unless otherwise mentioned and are color coded.

Supplemental Figure 3

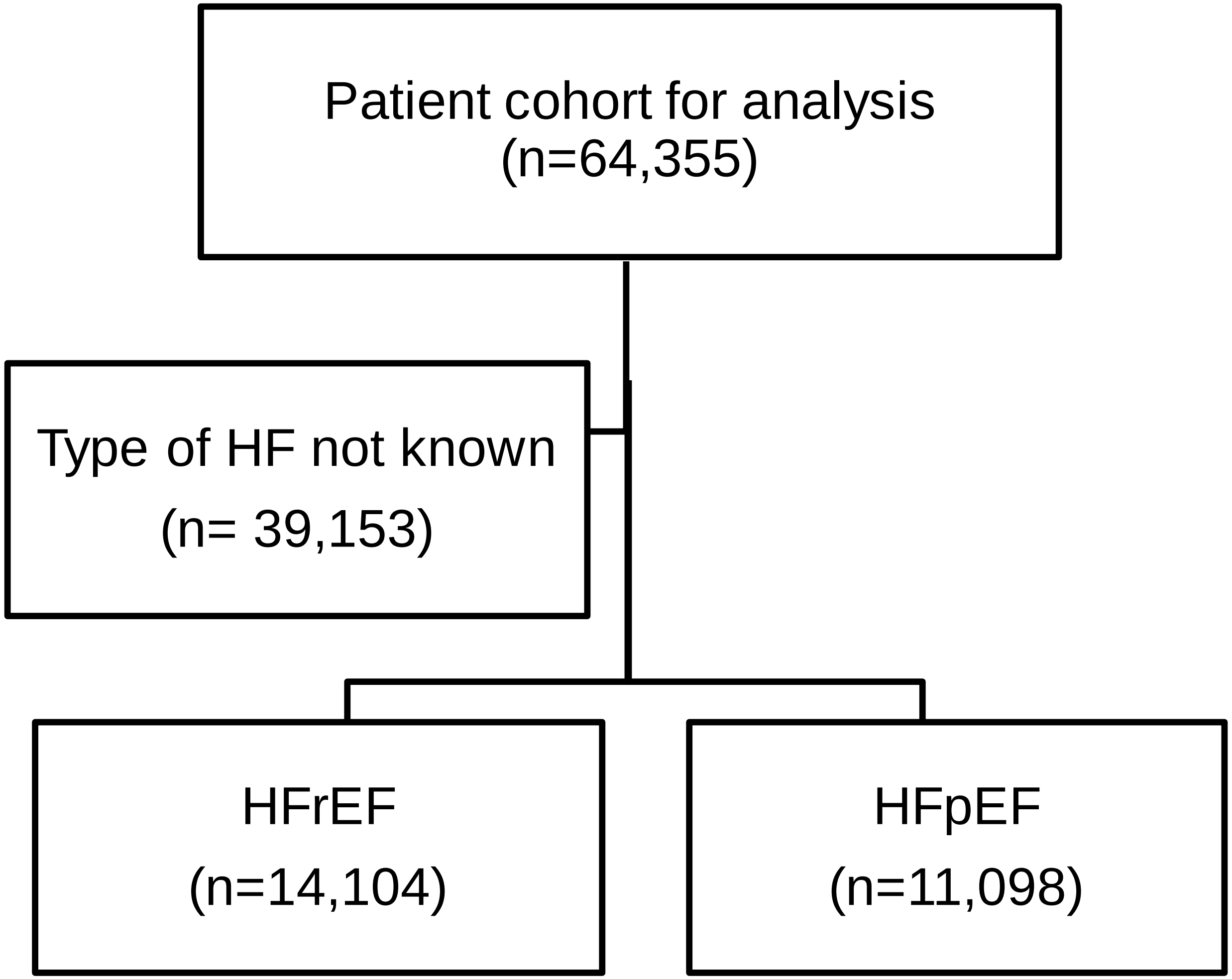

Supplemental Figure 4

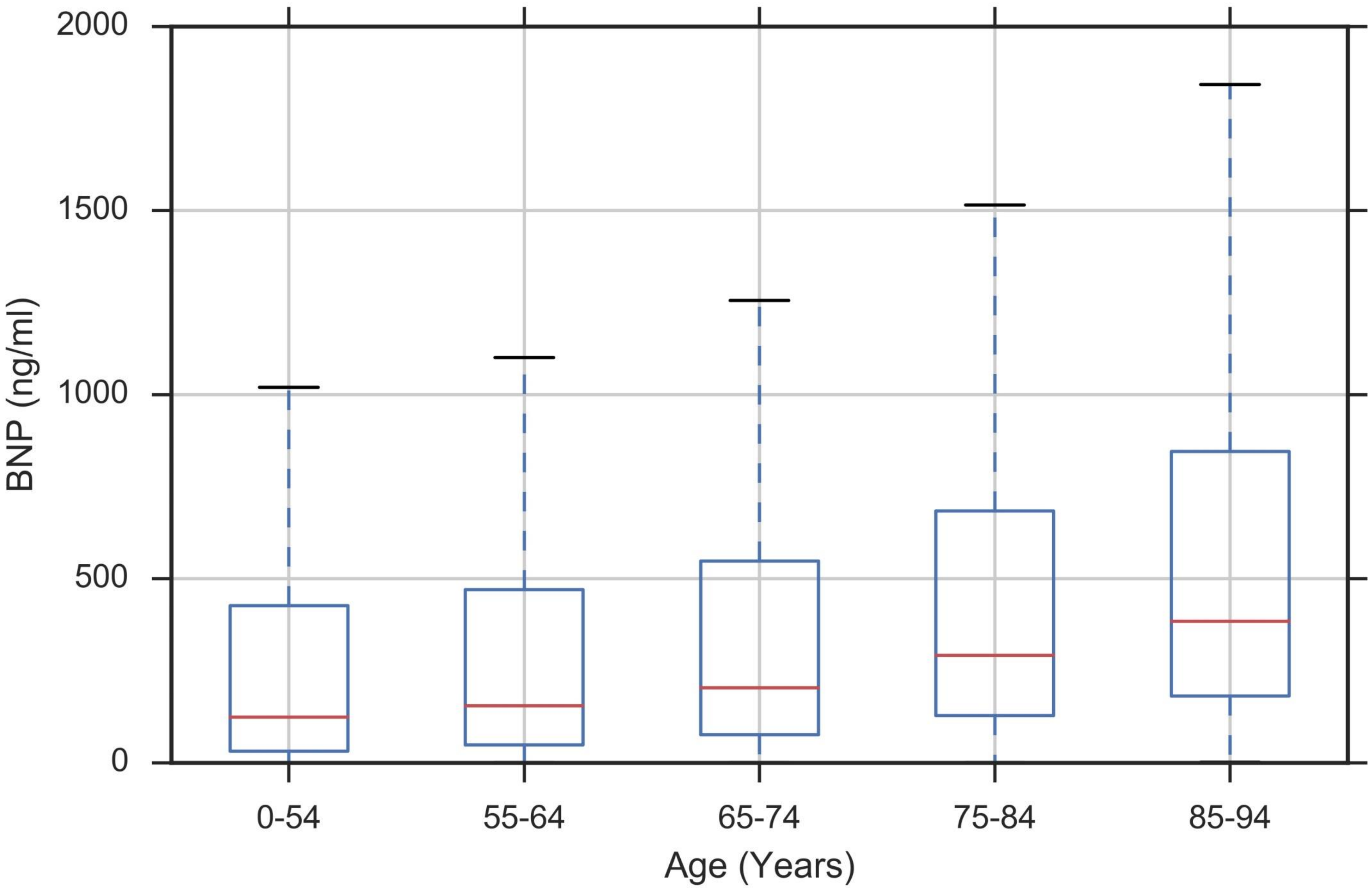

Supplemental Figure 5

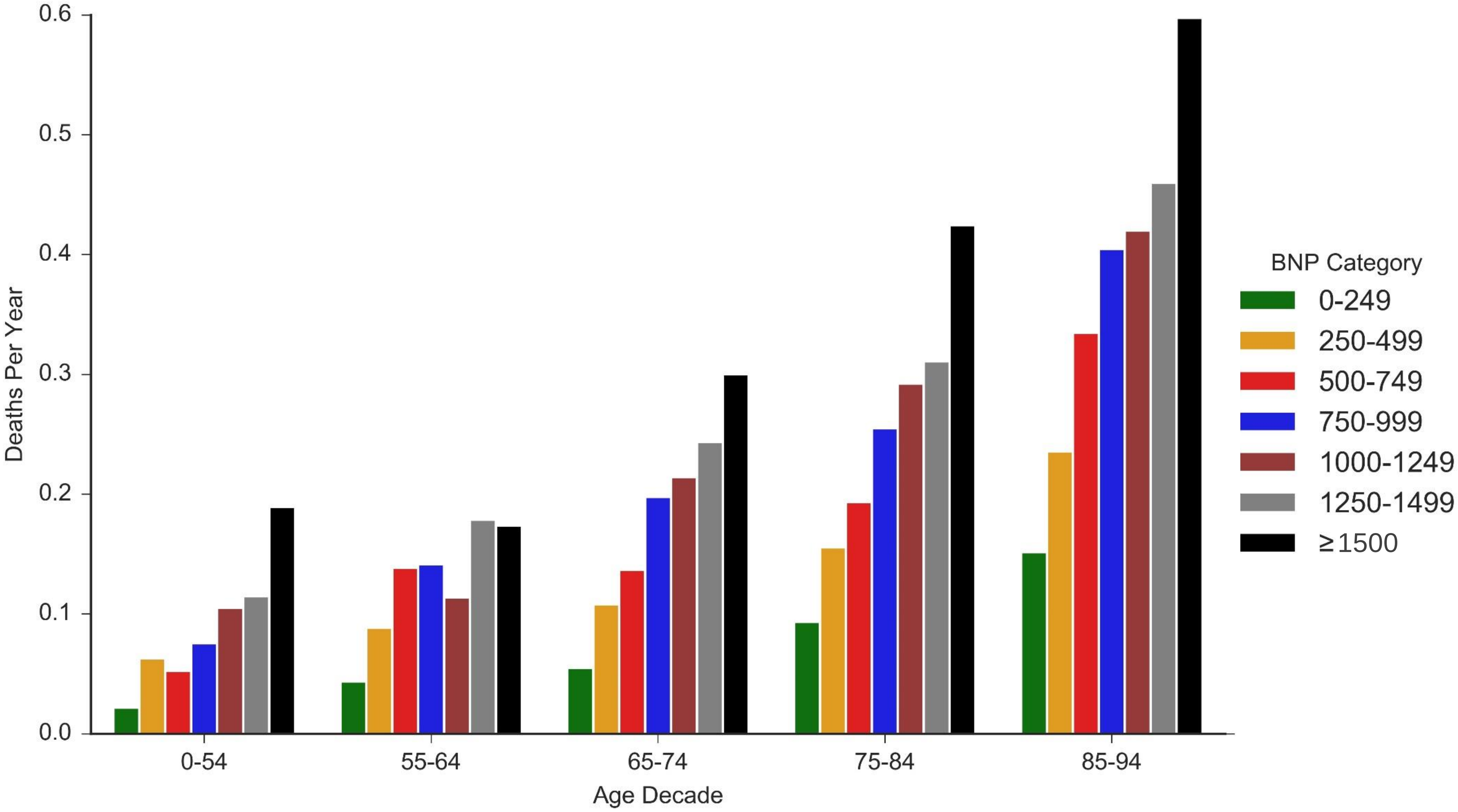

Supplemental Figure 6

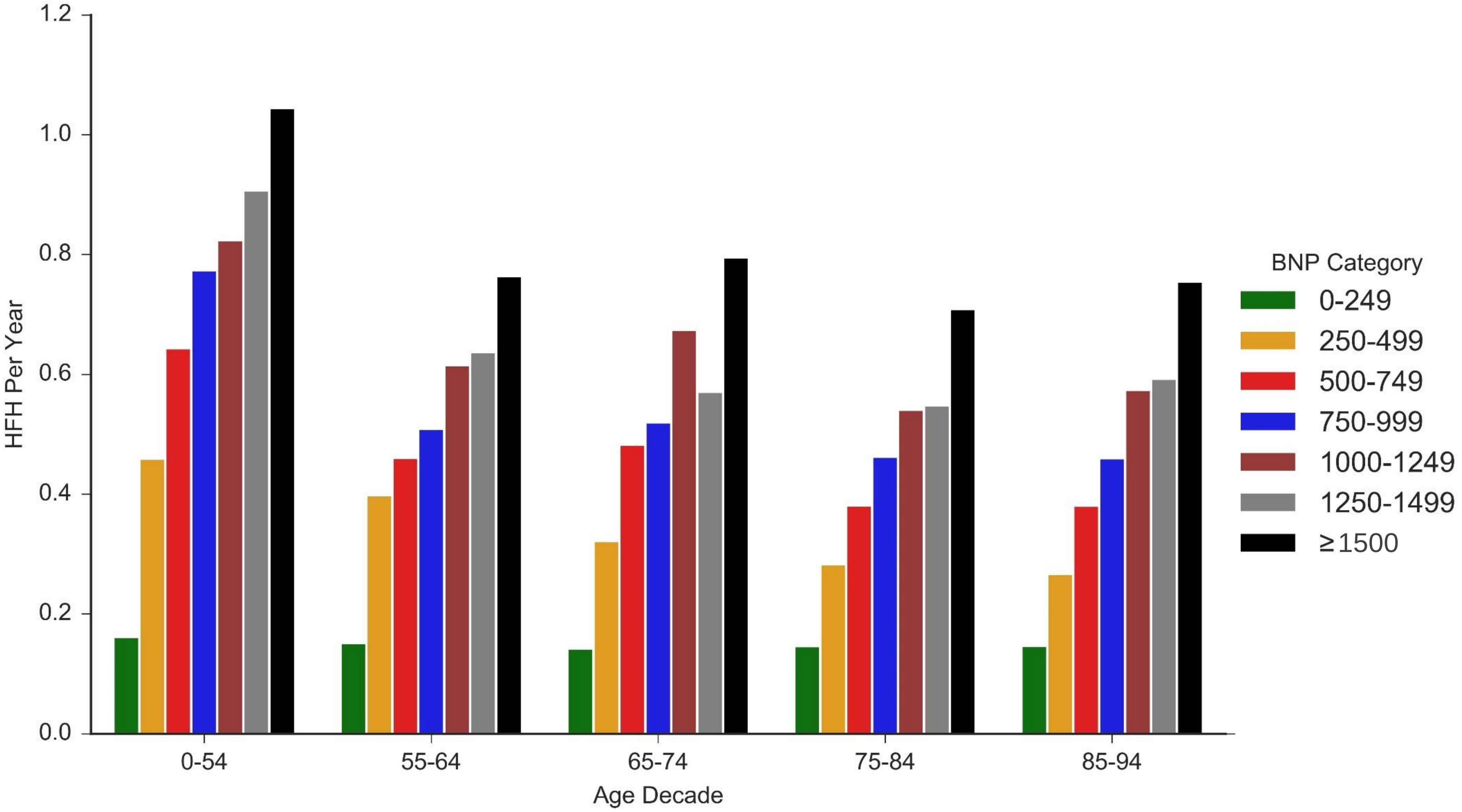

Supplement: Supplementary file 1 — Figure S1: Consort Diagram. Figure S2: 1 year mortality rates and heart failure hospitalization rates (HFH) rates across BNP levels with and without previous HFH (pHFH). This analysis examines the statistical difference at each BNP range between mortality with vs without pHFH (black bars) and between HFH with and without pHFH (red bars). For comparison of mortality between with‐ and without‐ pHFH, P‐values are <.001, unless otherwise mentioned and are color coded (black type). For comparison of HFH between with‐ and without‐ pHFH, all P‐values are <.001. For comparisons between each incremental BNP level, P‐values <.001, unless otherwise mentioned and are color coded (black type for mortality, red type for HFH. Figure S3: Consort Diagram for HFpEF vs HFrEF Cohort sub‐analysis Figure S4: Correlations between age and BNP levels Figure S5: Heart failure hospitalizations (HFH) rates stratified by age and BNP levels Figure S6: Mortality rates stratified by age and BNP levels [file CLC-43-1501-s001.pdf]
