# Supplementary material for: Prognostic value of brain natriuretic peptide vs history of heart failure hospitalization in a large real‐world population
Source: Clin Cardiol. 2020 Sep 19;43(12):1501–10. doi: 10.1002/clc.23468 (PMC7724209; doi:10.1002/clc.23468)
Supplement: Supplementary file 2 — Table S1: Patient characteristics Table S2: P‐values for each incremental BNP group comparison. Table S3: Risk ratios for adjusted BNP across different groups. Table S4: Estimated HF hospitalization rates and mortality rates from HF studies Table S5: Billing codes used to identify diagnosis for heart failure Table S6: Diagnoses Codes Used to Identify Comorbidities Table S7: Diagnoses Codes Used to Identify Death Table S8: Illustration of panel names and test names associated with BNP, NT‐proBNP for top 30 occurrences [file CLC-43-1501-s002.docx]

Supplemental Table 1: Patient Characteristics

| **BNP range (**pg/mL**)/**  **Comorbidities*** | **All** | **0-249** | **250-499** | **500-749** | **750-999** | **1000-1249** | **1250-1499** | **≥ 1500** | **P-value^†^** |
| --- | --- | --- | --- | --- | --- | --- | --- | --- | --- |
| **N** | 64,355 | 31,492 | 12,704 | 6,197 | 3,529 | 2,244 | 1,596 | 6,593 |  |
| Age | 74.3±11.7 | 72.1±12.1 | 76.6±10.5 | 76.8±10.7 | 76.8±10.9 | 76.5±11.1 | 76.4±11.3 | 75.9±11.3 | <0.001 |
| Gender (F) | 49.3% | 52.4% | 50.6% | 49.4% | 47.9% | 47.9% | 46.8% | 47.4% | <0.001 |
| AIDS/HIV | 0.3% | 0.4% | 0.3% | 0.3% | 0.3% | 0.2% | 0.2% | 0.2% | 0.113 |
| Alcohol abuse | 4.5% | 5.1% | 3.7% | 4.3% | 4.1% | 3.3% | 3.3% | 4.1% | <0.001 |
| Cancer | 5.8% | 5.8% | 5.7% | 5.6% | 6.0% | 6.1% | 6.1% | 6% | 0.678 |
| Cardiac Arrhythmias | 64.3% | 57.1% | 72.2% | 73.4% | 71.3% | 71.3% | 71.3% | 67.3% | <0.001 |
| Chronic pulmonary disease | 31.1% | 31.3% | 31.3% | 30.5% | 30.6% | 31.4% | 31.4% | 30.3% | 0.606 |
| Coronary Artery Disease | 57.1% | 52.9% | 59.5% | 60.2% | 62.3% | 62.1% | 62.1% | 63.4% | <0.001 |
| Anemia | 21.7% | 19.3% | 22.4% | 23.0% | 23.6% | 24.3% | 24.3% | 27.6% | <0.001 |
| Diabetes | 47.6% | 48.3% | 46.7% | 45.7% | 47.2% | 45.7% | 45.7% | 49.3% | <0.001 |
| Drug abuse | 5.3% | 5.9% | 5.0% | 4.9% | 4.3% | 4.5% | 4.5% | 4.3% | <0.001 |
| Fluid and electrolyte disorders | 29.7% | 26.5% | 29.9% | 31.8% | 32.6% | 33.9% | 33.9% | 38.9% | <0.001 |
| Hypertension | 88.1% | 88.2% | 88.2% | 88.2% | 88.1% | 87.7% | 87.7% | 87.7% | 0.778 |
| Hypothyroidism | 24.9% | 24.7% | 26.0% | 25.6% | 23.8% | 24.5% | 24.5% | 23.6% | 0.002 |
| Liver disease | 6.6% | 7.3% | 5.9% | 5.6% | 4.7% | 5.7% | 5.7% | 6.5% | <0.001 |
| Obesity | 25.1% | 31.5% | 22.2% | 19.3% | 18.0% | 17.2% | 17.2% | 14.0% | <0.001 |
| Neurological disorders | 25.2% | 27.1% | 23.9% | 23.0% | 22.7% | 24.2% | 24.2% | 23.3% | <0.001 |
| Peptic ulcer disease | 3.7% | 3.5% | 3.9% | 3.6% | 3.8% | 3.8% | 3.8% | 4.1% | 0.303 |
| Peripheral vascular disease | 30.3% | 27.4% | 35.2% | 34.6% | 31.2% | 32.3% | 33.8% | 35.9% | <0.001 |
| Pulmonary Circulation Disorder | 18.2% | 16.1% | 19.0% | 20.4% | 21.3% | 21.8% | 21.8% | 20.7% | <0.001 |
| Chronic Kidney Disease | 32.9% | 26.1% | 33.5% | 36.8% | 39.8% | 40.5% | 40.5% | 52.3% | <0.001 |
| Valvular disease | 35.4% | 30.1% | 39.1% | 41.3% | 40.8% | 43.4% | 43.4% | 40.6% | <0.001 |
| Weight loss | 8.1% | 6.6% | 8.3% | 9.7% | 8.7% | 10.0% | 10.0% | 12.3% | <0.001 |

**Comorbidities assessed per Quan 2005 and Moore 2017 methodology (Supplementary Table 2)*

†P-values test hypothesis if all BNP groups have same mean, and are calculated by Anova for continuous variables and Chi-square test for categorical variables

Supplemental Table 2: P-values for each incremental BNP group comparison.

| **BNP (pg/ml)/**  **Subgroups** | **0-249** | **250-499** | **500-749** | **750-999** | **1000-1249** | **1250-1499** | **≥ 1500** |
| --- | --- | --- | --- | --- | --- | --- | --- |
| **Mortality** | | | | | | | |
| All | - | <0.001 | <0.001 | <0.001 | 0.194 | 0.217 | <0.001 |
| No previous year HFH | - | <0.001 | <0.001 | <0.001 | 0.297 | 0.412 | 0.002 |
| Previous year HFH | - | <0.001 | 0.023 | 0.208 | 0.353 | 0.306 | 0.009 |
| HFrEF | - | <0.001 | <0.001 | 0.092 | 0.716 | 0.039 | 0.032 |
| HFpEF | - | <0.001 | 0.137 | 0.106 | 0.134 | 0.927 | 0.254 |
| **HFH** | | | | | | | |
| All | - | <0.001 | <0.001 | 0.156 | 0.005 | 0.323 | 0.007 |
| No previous year HFH | - | <0.001 | <0.001 | 0.023 | 0.176 | 0.842 | 0.038 |
| Previous year HFH | - | <0.001 | <0.001 | 0.835 | 0.006 | 0.256 | 0.045 |
| HFrEF | - | <0.001 | <0.001 | 0.950 | 0.075 | 0.473 | 0.545 |
| HFpEF | - | <0.001 | 0.081 | 0.929 | 0.028 | 0.415 | 0.667 |

Supplemental Table 3: Risk ratios for adjusted BNP across different groups. Adjusted BNP is increased by 75 pg/ml for a HFpEF patient, and increased by 4.1% for every unit increase in BMI above 25 units. Both BMI and EF were known in 3590 patients.

| **Adjusted BNP or NT-proBNP/4 (pg/ml)** | **HFH** | **Mortality** |
| --- | --- | --- |
|  | **RR[95% CI] p-value** | **RR[95% CI] p-value** |
| **0-249** | - | - |
| **250-499** | 1.8[1.5,2.3] <0.001 | 1.4[1.1,1.9] 0.015 |
| **500-749** | 1.9[1.5,2.4] <0.001 | 2.0[1.4,2.7] <0.001 |
| **750-999** | 2.4[1.8,3.3] <0.001 | 2.5[1.7,3.6] <0.001 |
| **1000-1249** | 2.3[1.6,3.2] <0.001 | 2.1[1.4,3.3] <0.001 |
| **1250-1499** | 2.1[1.5,3.0] <0.001 | 2.5[1.6,3.9] <0.001 |
| **≥ 1500** | 3.4[2.7,4.2] <0.001 | 3.8[2.9,5.1] <0.001 |

Supplemental Table 4: Estimated HF Hospitalization Rates and Mortality rates from HF studies

| **Study Name** | **NYHA Class Distribution (%)** | | | | **Relevant Inclusion**  **Criteria** | **Inclusion Criteria for BNP/NT-proBNP?** | | **HFH Event Rate (/pt-year)** | **Mortality Event**  **Rate (/pt-year)** |
| --- | --- | --- | --- | --- | --- | --- | --- | --- | --- |
|  | **I** | **II** | **III** | **IV** |  | **Yes/No** | **Details** |  |  |
| **CHAMPION (9)** | 0 | 0 | 100 | 0 | NYHA Class III HFH within 12 months | No | No BNP/NT-proBNP Information | 0.664 | 0.152 |
| **PARADIGM**  **(10)** | 5 | 69 | 25 | 1 | NYHA Class II-IV Elevated BNP/NT-proBNP Level | Yes | BNP>150, NT-proBNP>600 OR  BNP>100, NT-proBNP>400 with prior HFH within 12m | 0.136 | 0.088 |
| **SOCRATES**  **-REDUCED (11)** | 59 | | 41 | | NYHA Class II-IV LVEF < 45% HFH ( OR IV Diuretic) Congestion signs/symptoms  Elevated BNP/NT-proBNP | Yes | BNP ≥ 300, NT-proBNP ≥ 1000 (sinus rhythm) BNP ≥ 500, NT-proBNP ≥ 1600 (AF) | 0.754 | 0.141 |
| **REDHOT (12)** | 3 | 29 | 45 | 23 | Patients presenting to the ED treated for CHF | No | BNP ≥ 200 | - | 0.365 |
| **EMPHASIS-HF (13)** | 0 | 100 | 0 | 0 | NYHA Class II LVEF ≤ 30% HFH within 6 months OR Elevated BNP/NT-proBNP | Yes | BNP ≥ 250 pg/mL, NT-proBNP ≥ 500 pg/mL (men) OR ≥ 750 pg/mL (women) | 0.105 | 0.089 |
| **ATMOSPHERE (14)** | 0 | 62 | 36 | 2 | NYHA Class II-IV LVEF ≤ 35% Elevated BNP/NT-proBNP | Yes | BNP>150, NT-proBNP>600 OR  BNP>100, NT-proBNP>400 with prior HFH within 12m | 0.063 | 0.091 |
| **RELAX-AHF (15)** | 3 | 33 | 47 | 17 | NYHA Class I-IV Dyspnea, congestion Elevated BNP/NT-proBNP | Yes | BNP ≥ 350 pg/mL NT-proBNP ≥ 1400 pg/mL | 0.524 | 0.227 |
| **GUIDE-IT (16)** | 5 | 52 | 41 | 2 | HFrEF: LVEF ≤ 40% HFH (OR IV diuretic) within 12m Elevated BNP/NT-proBNP within 30 days | Yes | BNP ≥ 400 pg/mL OR NT-proBNP ≥ 2000 pg/mL | 0.495 | 0.138 |

Supplementary Table 5: Billing codes to identify diagnosis for heart failure

| ICD-9 CODE | ICD-9 CODE DESCRIPTION | ICD-10 CODE | ICD-10 CODE DESCRIPTION |
| --- | --- | --- | --- |
| 402.01 | Malignant hypertensive heart disease with heart failure | I110 | Hypertensive heart disease with heart failure |
| 402.11 | Benign hypertensive heart disease with heart failure | I110 | Hypertensive heart disease with heart failure |
| 402.91 | Unspecified hypertensive heart disease with heart failure | I110 | Hypertensive heart disease with heart failure |
| 404.01 | Hypertensive heart and chronic kidney disease, malignant, with heart failure and with chronic kidney disease stage I through stage IV, or unspecified | I130 | Hypertensive heart and chronic kidney disease with heart failure and stage 1 through stage 4 chronic kidney disease, or unspecified chronic kidney disease |
| 404.11 | Hypertensive heart and chronic kidney disease, benign, with heart failure and with chronic kidney disease stage I through stage IV, or unspecified | I130 | Hypertensive heart and chronic kidney disease with heart failure and stage 1 through stage 4 chronic kidney disease, or unspecified chronic kidney disease |
| 404.91 | Hypertensive heart and chronic kidney disease, unspecified, with heart failure and with chronic kidney disease stage I through stage IV, or unspecified | I130 | Hypertensive heart and chronic kidney disease with heart failure and stage 1 through stage 4 chronic kidney disease, or unspecified chronic kidney disease |
| 404.03 | Hypertensive heart and chronic kidney disease, malignant, with heart failure and with chronic kidney disease stage V or end stage renal disease | I132 | Hypertensive heart and chronic kidney disease with heart failure and with stage 5 chronic kidney disease, or end stage renal disease |
| 404.13 | Hypertensive heart and chronic kidney disease, benign, with heart failure and chronic kidney disease stage V or end stage renal disease | I132 | Hypertensive heart and chronic kidney disease with heart failure and with stage 5 chronic kidney disease, or end stage renal disease |
| 404.93 | Hypertensive heart and chronic kidney disease, unspecified, with heart failure and chronic kidney disease stage V or end stage renal disease | I132 | Hypertensive heart and chronic kidney disease with heart failure and with stage 5 chronic kidney disease, or end stage renal disease |
| 428.0 | Congestive heart failure, unspecified | I509 | Heart failure, unspecified |
| 428.9 | Heart failure, unspecified | I509 | Heart failure, unspecified |
| 428.1 | Left heart failure | I501 | Left ventricular failure |
| 428.20 | Systolic heart failure, unspecified | I5020 | Unspecified systolic (congestive) heart failure |
| 428.21 | Acute systolic heart failure | I5021 | Acute systolic (congestive) heart failure |
| 428.22 | Chronic systolic heart failure | I5022 | Chronic systolic (congestive) heart failure |
| 428.23 | Acute on chronic systolic heart failure | I5023 | Acute on chronic systolic (congestive) heart failure |
| 428.30 | Diastolic heart failure, unspecified | I5030 | Unspecified diastolic (congestive) heart failure |
| 428.31 | Acute diastolic heart failure | I5031 | Acute diastolic (congestive) heart failure |
| 428.32 | Chronic diastolic heart failure | I5032 | Chronic diastolic (congestive) heart failure |
| 428.33 | Acute on chronic diastolic heart failure | I5033 | Acute on chronic diastolic (congestive) heart failure |
| 428.40 | Combined systolic and diastolic heart failure, unspecified | I5040 | Unspecified combined systolic (congestive) and diastolic (congestive) heart failure |
| 428.41 | Acute combined systolic and diastolic heart failure | I5041 | Acute combined systolic (congestive) and diastolic (congestive) heart failure |
| 428.42 | Chronic combined systolic and diastolic heart failure | I5042 | Chronic combined systolic (congestive) and diastolic (congestive) heart failure |
| 428.43 | Acute on chronic combined systolic and diastolic heart failure | I5043 | Acute on chronic combined systolic (congestive) and diastolic (congestive) heart failure |

Supplementary Table 6: Diagnoses Codes Used to Identify Comorbidities

| Comorbidities | ICD-9-CM | ICD-10 |
| --- | --- | --- |
| Acquired immune deficiency syndrome | 042.x–044.x | B20 |
| Cancer | 196.x–199.x, 200.x–202.x, 203.0, 238.6 | C77.x–C80.x, C7B.x, C81.x–C85.x, C86.x, C88.x, C90.x, C96.x, D47.Z9 |
| Cardiac Arrhythmias | 426.0, 426.7, 426.9, 426.10, 426.12, 426.13,  427.0-427.4, 427.6-427.9, 7850, 9960.1,9960.4, V450, V533 | I44.1-I44.3, I45.6, I45.9, I47-I49, R00.0, R00.1, R00.8, T821, Z450, Z950 |
| Chronic Pulmonary Disease | 490 -496, 500-505,506.4 | J40.x–J47.x, J60.x–J67.x, J68.4 |
| Coronary artery disease (Chronic ischemic heart disease) | 414 | I25 |
| Deficiency Anemia | 280.1–280.9, 281 | D50.1, D50.8, D50.9, D51-D53, D63, D64.9 |
| Diabetes | 250.0–250.3, 250.4–250.9 | E08.0, E08.1, E09.0, E09.1, E09.9, O24.0-O24.3, O24.8-O24.9, E10.0-E10.9, E11.0-E11.9, E12.0-E12.9, E13.0-E13.9, E14.0-E14.9, P70.2 |
| Drug Abuse | 292, 304, 305.2–305.9, V6542 | F11-F16, F18, F19, F55, O9932 |
| Fluid and Electrolyte Disorder | 2536, 276 | E86, E87 |
| Hypertension | 401.x, 402.x–405.x | I10.x, I11.x–I13.x, I15.x, O10.x, O11.x, O16.x |
| Hypothyroidism | 240.9, 243.x, 244.x, 246.1, 246.8 | E00.x–E03.x, E89.0 |
| Liver Disease | 070.22, 070.23, 070.32, 070.33, 070.44, 070.54, 070.6, 070.9, 456.0–456.2, 570.x, 571.x, 572.2–572.8, 573.3, 573.4, 573.8, 573.9, V42.7 | B18.x, I85.x, K70.x, K72.x–K74.x, K76.0, K76.2–K76.9, Z94.4 |
| Obesity | 278.0 | E66.x, O99.2x, R939, Z68.3x-Z68.5x |
| Other neurological disorders | 331.9, 332.0, 332.1, 333.4, 333.5, 333.92, 334.x–335.x, 336.2, 340.x, 341.x, 345.x, 348.1, 348.3, 780.3, 784.3 | E75.x, G10.x–G13.x, G20.x–G21.x, G24.x, G25.4, G25.5, G25.81, G31.2, G31.8, G31.9, G32.x, G35.x–G37.x, G40.x, G47.x, G80.0, G89.0, G91.x, G93.1, G93.4, G93.8, G93.9, G94, R41.0, R41.82, R47.01, R56.x |
| Peptic ulcer disease excluding bleeding | 531.7, 531.9, 532.7, 532.9, 533.7, 533.9, 534.7, 534.9 | K25.4-K25.7, K25.9, K26.4-K26.7, K26.9, K27.4-K27.7, K27.9, K28.4-K28.7, K28.9 |
| Peripheral Vascular Disorder | 093.0, 437.3, 440.x, 441.x, 443.1– 443.9, 447.1, 557.1, 557.9, V43.4 | I70.x, I71.x, I72.x, I73.1, I73.8, I73.9, I77.1, I79.0, I79.1, I79.8, K55.1, K55.8, K55.9, Z95.8 |
| Pulmonary Circulation Disorders | 415.0, 415.1, 416.x, 417.0, 417.8, 417.9 | I26.x, I27.x, I28.9 |
| Renal Failure | 403.01, 403.11, 403.91, 404[019][23], 585, 586, 5880, V420, V451, V56 | N18, N19, Z49.0, Z49.3, Z91.15, Z94.0, Z99.2 |
| Valvular Disease | 093.2, 394.x–397.x, 424.x, 746.3–746.6, V42.2, V43.3 | A52.0, I05.x–I08.x, I09.1, I09.8, I34.x–I39.x, Q23.0–Q23.3, Z95.2–Z95.4 |
| Weight Loss | 260.x–263.x, 783.2, 799.4 | E40.x–E46.x, R63.4, R63.6 |

- *ICD-10 codes based on Moore BJ, White S, Washington R, Coenen N, Elixhauser A. Identifying increased risk of readmission and in-hospital mortality using hospital administrative data: The AHRQ Elixhauser Comorbidity index. Med Care. 2017 Jul; 55(7):698-705 and HCSRN: Health care systems research network* <http://hcsrn.org/sas/standard_macros.sas>
- *ICD-9 codes based on Quan et. al. Coding Algorithms for Defining Comorbidities in ICD-9-CM and ICD-10 Administrative Data. Med Care 2005;43: 1130–1139*

Supplementary Table 7: Diagnoses Codes Used to Identify Death

| **Death recorded in administrative claims** | **ICD-9-CM** | **ICD-10** |
| --- | --- | --- |
| **Sudden death** | 798x.x | R95.x, R96.x, R98.x |
| **Other death** |  | G9382, I46.1x, O95-7.x, P95.x, S06[0124569]X[78][SAD], S063[0-8][78][ADS], S068[129][78][SAD], Y355 |

Supplementary Table 8: Illustration of panel names and test names associated with BNP, NT-proBNP for top 30 occurrences

|  | **Panel Name** | **Test Name** | **Patients** |
| --- | --- | --- | --- |
| **1** | BNP | BNP | 4702 |
| **2** | BNP, NT pro | BNP NT Pro | 4196 |
| **3** | pro-BNP | pro-BNP | 3288 |
| **4** | B-Type Natriuretic Peptide, NT Pro | Natriuretic Peptide | 2923 |
| **5** | BNP B-type Natriuretic Peptide | BNP | 2703 |
| **6** | B-Type Natriuretic Peptide | B-Type Natriuretic Peptide | 2576 |
| **7** | BNP B-TYPE NATRIURETIC PEPTIDE | B-Type Natriuretic Peptide | 2550 |
| **8** | BNP: B-Natriuretic Peptide | BNP | 1714 |
| **9** | B-Type Natriuretic Peptide | BNP | 1462 |
| **10** | B TYPE NATRIURETIC$PEPTIDE (BNP) | B TYPE NATRIURETIC$PEPTIDE (BNP) | 1414 |
| **11** | B-TYPE NAT.PEPTIDE - BNP | Pro B Type BNP | 1394 |
| **12** | BNP - B TYPE NATRIURETIC PEPTIDE | B TYPE NATRIURETIC$PEPTIDE (BNP) | 1343 |
| **13** | NT-proBNP | NT-proBNP | 1193 |
| **14** | B-TYPE NATRIURETIC PEPTID | B-TYPE NATRIURETIC PEPTID | 1184 |
| **15** | BNP | B-Natriuretic Peptide | 1124 |
| **16** | B TYPE NATRIURETIC PEPTIDE (BNP) | B TYPE NATRIURETIC PEPTIDE (BNP) | 1116 |
| **17** | * BNP | BNP | 1026 |
| **18** | B TYPE NATRIURETICPEPTIDE BNP | B TYPE NATRIURETICPEPTIDE | 1006 |
| **19** | proBNP | PROBNP | 972 |
| **20** | B-TYPE NATRIURETIC PEPTIDE | BNP | 831 |
| **21** | B-Type Natriuretic Peptide ( BNP ) - 10013 | B-Type Natriuretic Peptide | 798 |
| **22** | BNP (B Natriuretic Peptide) | B NATRIURETIC PEPTIDE | 754 |
| **23** | NT-PRO BNP | NT-PRO BNP | 743 |
| **24** | B-Type Natriuretic Peptide | B TYPE NATRIURETIC PEPTIDE (BNP) | 732 |
| **25** | B-Natriuretic Peptide | B-Natriuretic Peptide | 710 |
| **26** | BNP B-NATRIURETIC PEPTIDE | B-Natriuretic Peptide | 699 |
| **27** | NTProBNP | NTProBNP | 691 |
| **28** | B-type natriuretic peptide (BNP) | BNP | 619 |
| **29** | BNP - B TYPE NATRIURETIC PEPTIDE | B-Type Natriuretic Peptide | 605 |
| **30** | BNP-Pro | BNP-Pro* | 594 |
|  |  |  |  |
